# 1,25-Dihydroxyvitamin D3 Treatment Delays Cellular Aging in Human Mesenchymal Stem Cells while Maintaining Their Multipotent Capacity

**DOI:** 10.1371/journal.pone.0029959

**Published:** 2012-01-05

**Authors:** Barbara Klotz, Birgit Mentrup, Martina Regensburger, Sabine Zeck, Jutta Schneidereit, Nicole Schupp, Christian Linden, Cornelia Merz, Regina Ebert, Franz Jakob

**Affiliations:** 1 Orthopedic Center for Musculoskeletal Research, University of Würzburg, Würzburg, Germany; 2 Institute of Pharmacology and Toxicology, University of Würzburg, Würzburg, Germany; 3 Institute for Virology and Immunobiology, University of Würzburg, Würzburg, Germany; University of Padova, Italy

## Abstract

1,25-dihydroxyvitamin D3 (1,25D3) was reported to induce premature organismal aging in *fibroblast growth factor-23* (*Fgf23*) and *klotho* deficient mice, which is of main interest as 1,25D3 supplementation of its precursor cholecalciferol is used in basic osteoporosis treatment. We wanted to know if 1,25D3 is able to modulate aging processes on a cellular level in human mesenchymal stem cells (hMSC). Effects of 100 nM 1,25D3 on hMSC were analyzed by cell proliferation and apoptosis assay, β-galactosidase staining, VDR and surface marker immunocytochemistry, RT-PCR of 1,25D3-responsive, quiescence- and replicative senescence-associated genes. 1,25D3 treatment significantly inhibited hMSC proliferation and apoptosis after 72 h and delayed the development of replicative senescence in long-term cultures according to β-galactosidase staining and *P16* expression. Cell morphology changed from a fibroblast like appearance to broad and rounded shapes. Long term treatment did not induce lineage commitment in terms of osteogenic pathways but maintained their clonogenic capacity, their surface marker characteristics (expression of CD73, CD90, CD105) and their multipotency to develop towards the chondrogenic, adipogenic and osteogenic pathways. In conclusion, 1,25D3 delays replicative senescence in primary hMSC while the pro-aging effects seen in mouse models might mainly be due to elevated systemic phosphate levels, which propagate organismal aging.

## Introduction

The biologically active metabolite of vitamin D, 1,25-dihydroxyvitamin D3 (1,25D3) is synthesized from precursors by sequential hydroxylations in the liver (25-hydroxylase) and kidney (1α-hydroxylase) [1]–[2]. 1,25D3 is involved in calcium and phosphate homeostasis through its effects on target organs such as intestine, kidney, parathyroid gland and bone [3]–[4]. There is also some evidence for a close association between hypervitaminosis D and accelerated aging in mice models [5]. Combined binding of the phosphatonin FGF23 and the longevity-associated gene product Klotho to the FGF receptor type 1 exerts FGF23 specific signaling [6]. Both, *Fgf23*-deficient (*Fgf23^−/−^*) and *klotho* (*kl^−/−^*) deficient mice, show a similar phenotype displaying growth retardation, growth plate abnormalities, highly elevated serum phosphate and serum 1,25D3 levels, infertility, arteriosclerosis, premature aging and a shortened lifespan [7]–[8]. Ablation of the vitamin D activation pathway by additional knockout of the *1α-hydroxylase* gene reverses anomalies in *Fgf23^−/−^* mice [9] and genetic inactivation of *1α-hydroxylase* gene in *kl^−/−^*/*1a(OH)ase^−/−^* mice reversed or abated the typical features observed in *kl^−/−^* mice [10]. Furthermore it was demonstrated that in *Fgf23^−/−^* mice with a non-functioning vitamin D receptor (VDR) the bone, mineral and glucose homeostasis could be rescued [11]. Therefore, interventions affecting the vitamin D responsive signal transduction seem to be closely linked to aging phenomena [5]. In contrast to these results, which linked VDR-dependent signaling to premature aging, it has been recently shown that VDR deficient mice develop premature aging phenomena, indicating that VDR-signaling might have anti-aging effects [12].

Mammalian aging is a complex biological process that can be defined as a progressive deterioration of physiological functions, as a decline of the functionality and regenerative capacity of all tissues and organs. It is accompanied by age-related diseases like arteriosclerosis, dementia, and osteoporosis [13]. Monogenetic mouse models and diseases in humans indicate that besides other mechanisms DNA and protein damage accumulation is associated with premature aging. Oxidative stress caused by reactive oxygen species (ROS) induces damage of the genome and proteome and promotes aging [14]–[15]. An imbalance between ROS production and detoxification leads to an increase of the ROS induced damage. The neutralization of ROS by a series of antioxidative enzymes and small molecules, e. g. superoxide dismutases or glutathione peroxidases, is an important segment of detoxification which also modulates the aging process [16].

Bone marrow derived human mesenchymal stem cells (hMSC) are multipotent and can give rise to mesenchymal tissues like bone, cartilage, and fat. They are a principal source of regeneration and healing and may be valuable tools for cell based regenerative therapies [17]–[18].

The aim of the present study was to characterize the effect of 1,25D3 on aging processes in hMSC. This was investigated in a series of experiments including e.g. RT-PCR analysis of quiescence- and senescence-associated genes, proliferation pace and ROS accumulation. Our hypothesis was that 1,25D3 is not a pro-aging compound at the cellular level and after having performed pilot studies we extended the hypothesis in that it might even delay cellular senescence.

## Results

### VDR immunocytochemistry and expression of 1,25D3 responsive genes in hMSC

**Figure 1:**
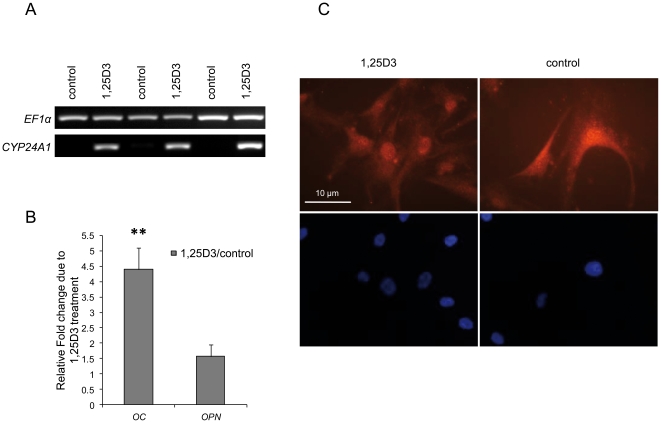
1,25D3 response in hMSC. **A/B** 1,25D3 stimulation of hMSC resulted in a strong increase of *CYP24A1* expression in all three donors. Control cells showed no *CYP24A1* expression, therefore densitometric quantification was not possible (A). The induction of *OC* expression is considerable and significant (**, p<0.01, student's t-test), the *OPN* expression is less intense. Densitometric quantification of *OC* and *OPN* expression was performed. For each donor the Fold Change was calculated by comparing the expression of 1,25D3 treated cells with control cells. The results were obtained from three independent donors and are shown as mean+SEM (B). **C** Immunostaining of VDR in hMSC after stimulation with 1,25D3 for 24 h. Stimulated cells show an increased localization of VDR in the nucleus, whereas the staining of the control cells is more diffuse (upper panel). Counterstaining with DAPI displays the localization of the nucleus (lower panel). One representative experiment is shown. Experiments were performed three times.

The effect of 1,25D3 on mRNA expression of responsive genes in hMSC was analyzed after stimulating cells from three donors for 24 h. To validate the method and to analyze regulation in hMSC we first amplified genes, known to be affected by 1,25D3 [19]. RT-PCR analysis resulted in an enhanced expression of 24-hydroxylase (*CYP24A1*), osteocalcin (*OC*) and osteopontin (*OPN*) in all three donors compared to unstimulated control cells (Fig. 1A/B). In summary, 1,25D3 modulates the expression of 1,25D3 responsive genes in hMSC.

Incubation of hMSC for 24 h with the VDR ligand 1,25D3 leads to a translocation of the liganded receptor into the nucleus, which was demonstrated by immunostaining with an antibody against human VDR (Fig. 1C). The intensive staining of a region overlapping with the nucleus, showed by DAPI staining, confirmed the nuclear translocation of liganded receptor in hMSC.

### Immunophenotype of hMSC treated with or without 1,25D3 at P1 and P3

The immunophenotype of hMSC was screened by surface antigen characterization using flow cytometry analysis (Fig. S1). To delineate possible phenotypic changes over time we analyzed P1 and P3 of 1,25D3 treated hMSC compared to untreated hMSC. Flow cytometry was not able to distinguish any differences between 1,25D3 stimulated hMSC and control hMSC at P1 and P3. Both, 1,25D3 treated hMSC and untreated cells, were negative for the hematopoietic markers CD34, CD45 and HLA-DR (Fig. S1, right panels), and positive for the mesenchymal markers CD73, CD90 and CD105 (Fig. S1, left panels). Fig. S1 shows 1,25D3 treated hMSC and control cells gated for CD73, CD90 and CD105 positivity. The expression of CD73, CD90 and CD105 of 1,25D3 treated hMSC and untreated cells was between 88.9% and 98.7%.

### Clonogenic capacity of 1,25D3 treated and untreated hMSC

1,25D3 stimulated and control hMSC were assayed for colony forming unit (CFU) frequency at P1 and P3, using cells from three donors (P3) and five independent donors (P1), respectively. No difference was observed between the two groups in terms of colony numbers at P1 and P3 (Fig. S2). The CFU frequency was highly donor-dependent and ranged from 318–1352 CFUs per 800 seeded hMSC at P1 and from 412–709 CFUs per 800 seeded cells at P3. Neither P1 nor P3 showed a significant correlation between 1,25D3 treatment and the number of CFUs. However, a nonsignificant decrease in CFUs could be observed in sets of cultures established at P3 in comparison to P1.

### Short term effects of 1,25D3 on proliferation and apoptosis in hMSC

**Figure 2:**
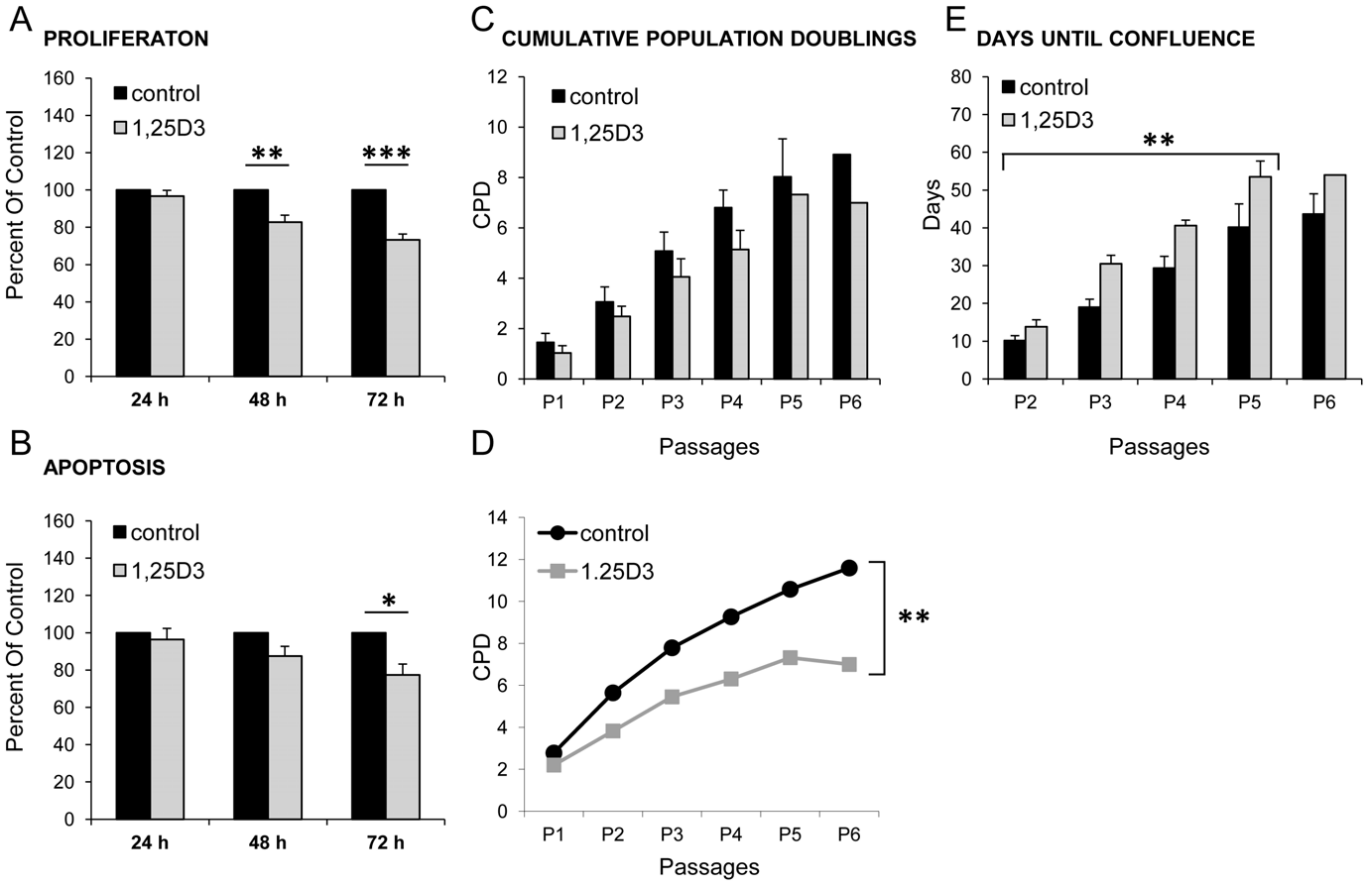
1,25D3 effects on proliferation, apoptosis and cumulative population doublings. **A/B** Measurement of proliferation capacity and apoptosis induction of hMSC from three different donors after stimulation with 1,25D3 for 24, 48 and 72 h. The proliferation rate was decreased time-dependently after 1,25D3 treatment (gray bars) compared to control cells (black bars), with significant effects after 48 h and 72 h (A). 1,25D3 stimulation showed the strongest reduction of apoptosis rates after 72 h 1,25D3 treatment (gray bars) compared to control cells (black bars, B). The results are shown as mean+SEM of three independent experiments, each normalized to its control and performed in triplicates. Cells from three different donors were used. (*, p<0.05; **, p<0.01;***, p<0.001, student's t-test). **C** The growth rates of cells were determined by population doublings at each subcultivation. Cumulative population doubling (CPD) was first determined for P2. Population doublings were observed for up to six cell passages. Persistent 1,25D3 supplementation resulted in a reduction of population doublings (gray bar), but the difference was not statistically significant. Independent experiments were performed using hMSC from several human donors. Control: P1, P2, n = 6; P3, P4, n = 5; P5, n = 3; P6, n = 2. 1,25D3: P1, P2, n = 6; P3, P4, n = 4; P5, P6, n = 1. **D** A representative example of CPDs of cells from one donor. 1,25D3 cultured cells exhibited lower CPDs compared to control hMSC (**, p<0.01, student's t-test). **E** The time required to reach subconfluence was determined. 1,25D3 treated cells (gray bars) needed more days until they reached subconfluence compared to control hMSC (black bars) (**, p<0.01, student's t-test). Independent experiments were performed using hMSC from several human donors. Control: P1, P2, P3, P4, n = 6; P5, n = 5; P6, n = 3. 1,25D3: P1, P2, P3, n = 6; P4, n = 5; P5, n = 4; P6, n = 1.

Stimulation of hMSC from three donors for 24, 48 and 72 h with 1,25D3 revealed similar effects on proliferation and apoptosis. 1,25D3 treatment of hMSC leads to a significant inhibition of cell proliferation after 48 h (17% reduction; **, p<0.01) and 72 h (27% reduction; ***, p<0.001) (Fig. 2A). Only a marginal inhibition of cell proliferation was noted after 24 h treatment. The apoptosis of hMSC was inhibited by 1,25D3 treatment after 48 h (12% reduction) and 72 h (23%; *, p<0.05), respectively. After 24 h only minor effects on apoptosis could be observed (Fig. 2B).

### Growth rate of hMSC permanently treated with 1,25D3

The proliferation capacities (cumulative population doublings, CPDs) of hMSC grown under permanent 1,25D3 treatment showed lower CPDs (gray bar) versus control cells (black bar) (Fig. 2C). The proliferation rate of control hMSC was somewhat higher but without any significance compared to 1,25D3 treated cells. The CPDs of hMSC from several different donors (as indicated in the legend) were analyzed for up to six passages (Fig. 2C). Figure 2D shows growth curves and their respective CPDs for one donor. 1,25D3 treated hMSC displayed significantly lower CPDs compared to control cells (**, p<0.01). Although there was a donor-related variability of the proliferation rate, 1,25D3 stimulation caused an obvious growth retardation and 1,25D3 stimulated cells needed significantly more days until they reached subconfluence (**, p<0.01) (Fig. 2E). The results are shown as mean from several donors (see figure legend for more information) and were consistent in all investigated donors.

### Morphological characteristics of 1,25D3 cultured hMSC

**Figure 3:**
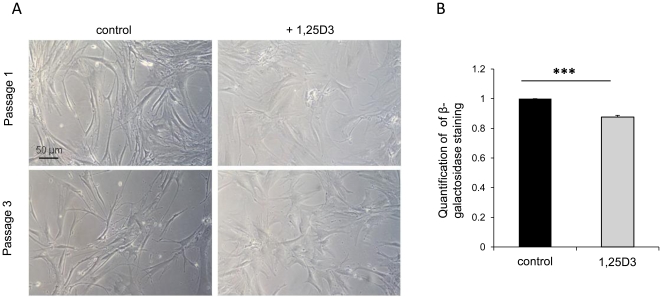
Morphological characteristics of 1,25D3 cultured hMSC and ß-galactosidase quantification. **A** Morphology of hMSC in P1 (upper row) and P3 (lower row) treated with 1,25D3 over three passages compared to control cells. 1,25D3 incubated hMSC lost their typical fibroblast-like features and showed a broadened and extended cellular morphology. Scale bar = 50 µm. **B** The induction of ß-galactosidase was significantly reduced in 1,25D3 cultured cells compared to control hMSC. Cells from three different donors were used and were cultured up to three passages. The results are shown as mean+SEM of three independent experiments, each normalized to its control. Each time five pictures of ß-galactosidase staining were analyzed using the AutMess tool of AxioVision Rel. 4.6 software. (***, p<0.001, student's t-test).

1,25D3 treatment induced obvious morphological changes in hMSC. Cells lost their typical fibroblast-like features, enlarged their original cell volume and developed broadened and extended shapes compared to untreated hMSC. No signs of spontaneous differentiation, e.g. towards the development of lipid droplets or mineralization induction could be observed (Fig. 3A).

### Retransformation of 1,25D3 stimulated hMSC

These 1,25D3 induced morphological changes in hMSC are partially reversible. After 1,25D3 treatment of hMSC over three passages cells were put back to normal medium (retransformed cells; r.c.). After two weeks the morphological changes were partially rescued (Fig. S3A). Some cells retained their 1,25D3 induced enlarged phenotype, some cells got their typical fibroblast-like features back. The cell number of control cells was nonsignificantly higher (average of three donors: cell number = 1.6×10^5^) compared to the cell number of 1,25D3 treated cells (average of three donors: cell number = 0.8×10^5^) at the same passages (Fig. S3B/C). The results are shown for three donors and the observed phenomena were consistent over all analyzed hMSC donors.

### Senescence-associated ß-galactosidase in 1,25D3 stimulated hMSC

The impact of 1,25D3 on cellular senescence was determined by senescence-associated ß-galactosidase staining in stimulated and unstimulated hMSC. Three different donors were cultivated with and without 1,25D3 for up to three passages. In stimulated hMSC (gray bar) a significant reduction of ß-galactosidase staining was observed compared to unstimulated cells (black bar) (p<0.001) (Fig. 3B). Furthermore we were able to follow up one donor for nine passages and compared 1,25D3 treated cells to control cells of P9. The significant effect, which was obvious at P3, was even more impressive in this late passage. 40 percent reduction of senescent hMSC could be counted (p<0.01) (data not shown).

### ROS accumulation in 1,25D3 treated hMSC

The accumulation of ROS in 1,25D3 supplemented cells compared to untreated hMSC after P1 and P3 was measured by flow cytometry. As shown in Fig. S5A, a slight and nonsignificant reduction of oxidative stress induction after treating hMSC for one passage with 1,25D3 was observed. As depicted in Fig. S5B, 1,25D3 treatment of hMSC for a period of three passages led to a significant increase in ROS formation (*, p<0.05).

### Effects of 1,25D3 treatment on the trilineage differentiation capacity of hMSC

**Figure 4:**
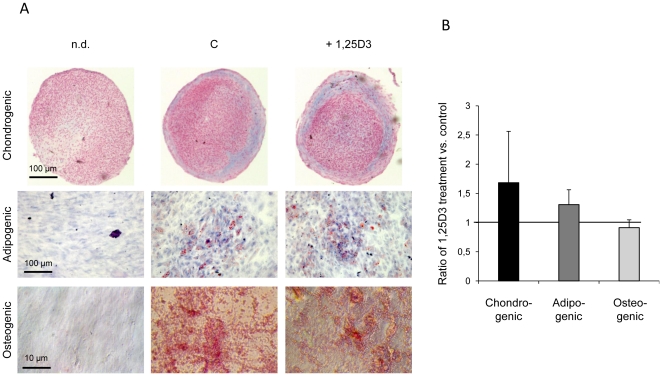
Trilineage differentiation capacity of 1,25D3 pretreated hMSC. **A** Chondrogenic, adipogenic and osteogenic differentiation of hMSC after 1,25D3 pretreatment over three passages. Alcian Blue staining of chondrogenic differentiated hMSC (upper panel), Oil Red O staining for intracellular lipid vesicles during adipogenic differentiation (middle panel) and Alizarin Red S staining of mineralized extracellular matrix after osteogenic differentiation (lower panel) are shown (n.d. = not differentiated; C = differentiated control; +1,25D3 = differentiated, cells pretreated for three passages with 1,25D3). The figure is representative for three independent experiments. The bar represents 100 µm and 10 µm, respectively as indicated. **B** Quantification of chondrogenic (black bar), adipogenic (dark gray bar) and osteogenic differentiation (light gray bar) after three passages 1,25D3 pretreatment in comparison to untreated cells. For each differentiation lineage the Fold Change was calculated by comparing the induction of differentiation of 1,25D3 pretreated cells with control cells. The results are shown as means of three independent experiments+SEM using three different preparations of hMSC. Each time 5 to 9 pictures of each differentiation experiment were analyzed using the AutMess tool of the software of AxioVision Rel. 4.6.

The maintenance of the multilineage capacity of 1,25D3 treated hMSC could be determined by subsequent chondrogenic, adipogenic and osteogenic differentiation in comparison to untreated controls. The induction of chondrogenesis, determined by Alcian blue staining of proteoglycans (Fig. 4A, upper panel), was quantified on digital images taken from chondrogenic pellets. 1,25D3 pretreatment increased the chondrogenic capacity of hMSC compared to cells grown without 1,25D3 supplementation, although values were not significant (Fig. 4B). Variability between the donors was observed. Two donors displayed almost the same chondrogenic induction between untreated differentiated cells and 1,25D3 pretreated hMSC, whereas one donor showed a strong enhanced induction of chondrogenesis after 1,25D3 pretreatment.

We also determined the adipogenic capacity of 1,25D3 pretreated cells versus untreated control hMSC. After adipogenic induction, oil droplet formation was found to a lesser extent in control hMSC than in 1,25D3 pretreated cells (Fig. 4A, middle panel). This difference was not statistically significant (Fig. 4B).

After two weeks under osteogenic conditions, 1,25D3 pretreated hMSC showed a slightly enhanced ALP staining compared to untreated cells (Fig. S4A, upper panel and B). This effect was reversed after four weeks of osteogenic culturing (Fig. S4A, lower panel and B), where both 1,25D3 pretreated and untreated hMSC displayed an almost equivalent amount of mineralization shown by Alizarin Red O staining (Fig. 4A, lower panel and B).

### Effects of 1,25D3 treatment on the expression of quiescence markers

**Figure 5:**
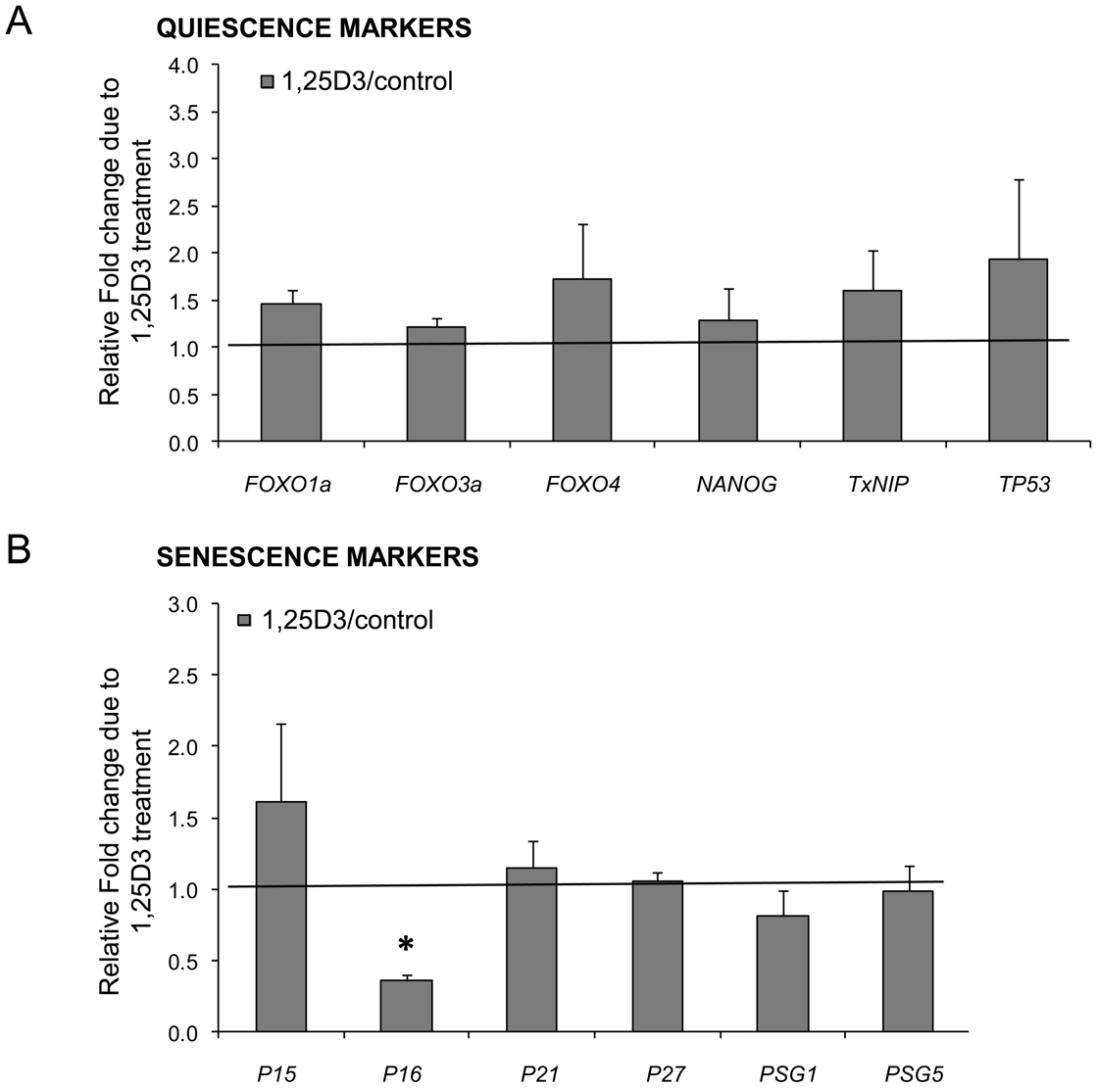
Effects of 1,25D3 treatment on quiescence- and senescence-associated genes. **A** Expression of quiescence markers and densitometric quantification of semiquantitative RT-PCR results of hMSC (P3) treated with or without 1,25D3. Cells from five different donors were used. For each donor the Fold Change was calculated by comparing the expression of 1,25D3 treated cells with control cells. The results are shown as mean+SEM. The gene expression levels of *FOXO1a*, *FOXO3a*, *FOXO4*, *NANOG*, *TxNIP*, *TP53* were induced by 1,25D3 treatment over 3 passages. (FOXO1a: forkhead box 1a, FOXO3a: forkhead box 3a, FOXO4: forkhead box 4, NANOG: Nanog homeobox, TxNIP: thioredoxin interacting protein, TP53: tumor protein p53). **B** Expression of senescence markers and densitometric quantification of semiquantitative RT-PCR results of hMSC treated with or without 1,25D3 over at least four passages. 1,25D3 treatment reduced *P16* expression (p<0.05) and induced *P15* expression in cells at P4 compared to control cells. *P27* and *P21* gene expression in P4 showed no (*P27*) or only very weak (*P21)* induction by 1,25D3 treatment. Densitometric quantification of the semi-quantitative PCR results revealed downregulation of *PSG1* and almost no change in the expression of *PSG5* at P4 in 1,25D3 stimulated hMSC. The Relative Fold change represents the factor of different gene expression levels in 1,25D3 treated hMSC versus control cells. Expression profile of senescence-associated genes was determined in P4. Cells from three different donors were used. For each donor the Fold Change was calculated by comparing the expression of 1,25D3 treated cells with control cells. The results are shown as mean+SEM. (*, p<0.05, student's t-test).

To investigate if 1,25D3 treatment over three passages had an impact on cellular quiescence, we analyzed the expression level of selected quiescence-associated genes. The gene expression of forkhead box O1 (*FOXO1a*) displayed a fold change of 1.5, forkhead box O3 (*FOXO3a*) was 1.2-fold up-regulated and forkhead box O4 (*FOXO4*) was 1.7-fold up-regulated in 1,25D3 stimulated hMSC at P3 compared to control cells. Nanog homeobox (*NANOG*) is slightly induced by 1,25D3 treatment and revealed a fold change of 1.3. Thioredoxin interacting protein (*TxNIP*) was induced in 1,25D3 treated cells and was 1.6 -fold upregulated. Tumor protein p53 (*TP53*) showed a 1.9-fold upregulation due to 1,25D3 treatment (Fig. 5A).

### Effects of 1,25D3 treatment on the expression of senescence markers

The gene expression of the senescence-associated genes cyclin-dependent kinase inhibitor 2B (*P15*), cyclin-dependent kinase inhibitor 2A (*P16*), cyclin-dependent kinase inhibitor 1A (*P21*) and proteasome 26S subunit, non-ATPase, 9 (*P27*) in hMSC cultured with or without 1,25D3 for four passages led to a significant 0.4-fold (p<0.05) downregulation of *P16* gene expression and to a nonsignificant 1.6-fold upregulation of *P15* expression compared to control cells. 1,25D3 treatment had no effect on *P27* expression and *P21* expression was only marginally induced (fold change = 1.2). Long-term stimulation of hMSC (P4) had also no significant effects on mRNA expression of the senescence-associated pregnancy specific beta-glycoproteins *PSG*s. We focused our analyses on *PSG1* and *PSG5* and could demonstrate that the gene expression of *PSG1* was slightly 0.8-fold downregulated in 1,25D3 treated cells in P4 while the expression of *PSG5* was unchanged (Fig. 5B).

## Discussion

Based on the knowledge of aging-promoting properties of unbalanced systemic 1,25D3 excess in animal models we analyzed cellular 1,25D3 effects on hMSC by cell proliferation and apoptosis assay, β-galactosidase staining, VDR and surface marker immunocytochemistry and RT-PCR of 1,25D3-responsive, quiescence- and replicative senescence-associated genes.

In order to validate the response of hMSC to 1,25D3 treatment, we first showed that 1,25D3 short-term stimulation modulates the responsive genes *CYP24A1*, *OC* and *OPN* in hMSC. Additionally, nuclear translocation of the liganded VDR receptor could be confirmed by immunostaining.

To characterize hMSC, which were stimulated with 1,25D3 for three or more passages, we investigated surface marker analysis, clonogenic capacity and cell morphology. The analysis of the surface marker expression of 1,25D3 stimulated hMSC did not change with respect to mesenchymal markers (CD73^+^, CD90^+^, CD105^+^) and hematopoetic markers (CD34^−^, CD45^−^, HLA-DR^−^) as analyzed by FACS in comparison to untreated cells. Moreover there was no loss of clonogenic capacity in 1,25D3 treated hMSC. 1,25D3 treated hMSC lost their typical fibroblast-like features, enlarged their original cell volume and developed broadened and extended shapes compared to untreated hMSC. When 1,25D3 was withdrawn in long term cultures the morphology only partially returned to the fibroblast like appearance.

In short-term time response experiments, stimulation of hMSC with 1,25D3 showed the strongest inhibitory effect on hMSC proliferation and apoptosis after 72 h. This might directly result from the interaction of 1,25D3 with its nuclear receptor nVDR and the modulation of gene transcription. Otherwise secondary effects could also be involved since 1,25D3 responsive genes might regulate downstream effects. The effect on hMSC however is different with respect to lineage commitment from e.g. that on promyelocytes where 1,25D3 induces monocytic differentiation together with inhibition of proliferation [20].

Numerous *in vitro* and *in vivo* studies have demonstrated that 1,25D3 inhibits proliferation of a wide range of cell types. In smooth muscle cells 1,25D3 inhibits endothelin-induced proliferation [21]–[22] and Gonzalez-Pardo *et al.* [23] mentioned the antiproliferative effects of 1,25D3 and the 1,25D3-analog TX527 on the growth of endothelial cells *in vitro* and *in vivo*. Our results demonstrate the ability of 1,25D3 to inhibit proliferation rate in hMSC compared to control cells. Human MSC grown under permanent 1,25D3 treatment for up to six passages showed lower CPDs compared to control cells and needed significantly more days until they reached subconfluence, suggesting that vitamin D signaling leads to a deceleration of proliferative processes, accompanied by changes of cell morphology and a delay of replicative senescence.

This could be confirmed by staining and quantification of ß-galactosidase, an accepted marker for cellular senescence [24], in 1,25D3 treated hMSC compared to control cells. The number of positive cells from P3 in three donors after staining for senescence-associated ß-galactosidase was compared. The amount of ß-galactosidase staining in 1,25D3 stimulated cells was significantly lower compared to control hMSC. Although the determination of ROS accumulation in 1,25D3 supplemented cells was significantly enhanced compared to control cells (P3) 1,25D3 stimulation had no impact on the induction of senescence in hMSC.

Although 1,25D3 is not capable of inducing robust commitment and differentiation towards the chondrogenic, adipogenic and osteogenic pathway, the stimulatory effect in the gut on calcium and phosphate absorption is generally well accepted as a pro-osteogenic and pro-mineralization effector. We determined the multilineage capacity of 1,25D3 pretreated cells compared to untreated control hMSC. There was no difference with respect to their capacity to differentiate towards one of these pathways. Moreover, there was even a tendency, although not significant, towards enhanced chondrogenic and adipogenic differentiation. It has been reported earlier that 1,25D3 stimulates chondrogenic differentiation via the membrane-associated MARRS-receptor by modulating IGF-1 production, which would be in line with our results [25]. We conclude from these results that long-term 1,25D3 treatment *in vitro* did not per se induce chondrogenic, adipogenic or osteogenic commitment and that hMSC maintain their multipotency even after long-term stimulation with 1,25D3.

The effect of reduced proliferation and changes in cell morphology raised the question regarding the biology of the resulting population. To find evidence for quiescence we analyzed the expression of known quiescence markers [26] as FOXO mRNAs and also *NANOG*, *TxNIP* and *TP53*. The forkhead box O (FOXO) proteins are activated in response to oxidative stress and up-regulate genes involved in ROS detoxification and cell-cycle arrest [27]–[28]. In five donors permanent 1,25D3 treatment leads to a modest up-regulation of *FOXO1a*, *FOXO3a* and *FOXO4*. Pierantozzi [29] could show that in hMSC Nanog homeobox (*NANOG*) expression is associated with quiescence and our data demonstrate a weak induction of *NANOG* expression in 1,25D3 stimulated cells in all donors. Furthermore the Thioredoxin interacting protein (*TxNIP*), a regulator of hematopoietic stem cell (HSC) quiescence under stress conditions [30], was induced in 1,25D3 treated cells. Finally also the tumor protein *TP53* (*P53*), which has an important role in the cellular response to DNA damage and plays also a critical role in regulating HSC [31], is up-regulated by 1,25D3. In summary we see a trend toward an enhanced expression of genes associated with cellular quiescence. This interpretation may also be fostered by the fact that 1,25D3 treatment attenuates cumulative population doublings without inducing senescence.

To follow up the impact of permanent 1,25D3 stimulation in hMSC we also analyzed the expression of selected genes associated with senescence. This analysis of senescence-associated genes like the cyclin-dependent kinase inhibitors 2B (*P15*), 2A (*P16*), 1A (*P21*) and the proteasome 26S subunit, non-ATPase, 9 (*P27*), which regulate the cell cycle of eukaryotic cells [32] surprisingly revealed a significant reduction of *P16* expression in permanently 1,25D3 stimulated cells. The senescence associated genes *P15*, *P21* and *P27* were not significantly affected in comparison to unstimulated controls. Furthermore also the pregnancy specific beta-glycoproteins (*PSG1, PSG5*), which are known to be markedly up-regulated during replicative senescence [33] were not induced by 1,25D3. Hence we have no evidence for an enhanced expression of senescence-associated genes in 1,25D3 stimulated MSC, but substantial evidence for the opposite effect.

In conclusion our data suggest that 1,25D3 does not induce premature aging phenomena or cellular senescence in hMSC. Our data are very much in line with the recently published phenotype of premature aging in *VDR* ^−/−^ mice. Besides the fact that vitamin D3 supplementation have been given to humans for at least 40 years without significant health problems our results are especially reassuring since is established as a basic therapy in osteoporosis prevention and treatment and is presently being discussed as a means of cancer prevention [34]. The preponderance of evidence from all readouts indicates that 1,25D3 does not promote aging in this important cell population for mesenchymal tissue regeneration but in contrast delays senescence development.

## Materials and Methods

### Chemicals

Chemicals were obtained from AppliChem GmbH (Darmstadt, Germany), Applied Biosystems Deutschland GmbH (Darmstadt, Germany), Invitrogen GmbH (Karlsruhe, Germany), Carl Roth GmbH (Karlsruhe, Germany), R&D Systems GmbH (Wiesbaden, Germany), Sigma-Aldrich GmbH (Schnelldorf, Germany) and GE Healthcare Europe GmbH (Freiburg, Germany) in p. A. quality. 1,25D3 was obtained from Sigma-Aldrich GmbH (Schnelldorf, Germany).

All experiments were performed using 1,25D3 in a concentration of 100 nM.

### Isolation and culture of hMSC

Media for cell culture and fetal bovine serum were purchased from PAA Laboratories GmbH (Linz, Austria).

Primary hMSC were isolated from femoral heads due to hip replacement therapies from 11 different donors and cultured as described [35]–[36]. Cells were plated for stimulation experiments in P1 with or without 100 nM 1,25D3 at a density of 5000 cells per cm^2^ and were cultivated until 80%–90% confluence at 37°C in 5% CO_2_ in expansion medium (DMEM Ham's F12 supplemented with 10% FCS, 1 U/ml penicillin, 100 µg/ml streptomycin and 50 µg/ml ascorbate). Experiments were performed upon approval by the Local Ethics Committee of the University of Würzburg and informed consent from each patient.

### Isolation of RNA and RT-PCR

Total RNA was isolated using the NucleoSpin® RNAII kit (Macherey-Nagel GmbH, Düren, Germany) according to the manufacturer's instructions. RNA was obtained from hMSC cultivated for 24 h with 100 nM 1,25D3.

Two micrograms of total RNA were reverse-transcribed with MMLV reverse transcriptase (Promega GmbH, Mannheim, Germany) in a volume of 25 µl. For amplification of selected genes 1 µl of cDNA was used as a template in a volume of 50 µl. The mixture contained 10× Reaction Buffer, 20 mM each of four dNTPs, 25 pmol each of primer and 1.25 U of Taq DNA Polymerase (Peqlab GmbH, Erlangen, Germany). RT-PCR conditions were as follows: 30 sec at 94 C, 30 sec at annealing temperature, 30 sec at 72 C. The used primer-sequences, annealing temperatures, MgCl_2_ concentrations, and the size of the PCR products are listed in Table 1. Transcript amounts were evaluated by gel electrophoresis and quantified by densitometry analysis as described previously [37] using the LTF Bio 1D software (LTF, Wasserburg, Germany). Values were obtained from three to five independent experiments and were normalized to EF1α transcripts serving as a reference. Afterwards mRNA amounts of analyzed genes were normalized on mRNA quantities of corresponding control. Then for each donor the Fold Change was calculated by comparing the expression of 1,25D3 treated cells with control cells. The results are shown as mean+SEM.

**Table 1:** Primers used for RT-PCR experiments.

Primer	Sequence 5′→3′	Product size (bp)	Cycles (n)	Tm (°C)	MgCl_2_ (mM)
EF-1-α for	AGGTGATTATCCTGAACCATCC	233	30	54	1.5
EF-1-α rev	AAAGGTGGATAGTCTGACTGTTG	233	30	54	1.5
P16 for	CTTCCTGGACACGCTGGT	138	35	55	2.0
P16 rev	GCATGGTTACTGCCTCTGGT	138	35	55	2.0
24OHASE for	TATGAGGCTTACGCCGAGTG	446	40	62	1.5
24OHASE rev	CCGCTTCCCTGAGTTGGATG	446	40	62	1.5
OP for	ACCCTTCCAAGTAAGTCCAA	400	40	58	2.5
OP rev	GTGATGTCCTCGTCTGTAGC	400	40	58	2.5
OC for	ATGAGAGCCCTCACACTCCTC	293	35	60	2.5
OC rev	GCCGTAGAAGCGCCGATAGGC	293	35	60	2.5
PSG1 for	TGAAGTCAGCCTTGGTTTGG	240	35	55	2.5
PSG1 rev	TTTCCCTCTATGGGCATCTC	240	35	55	2.5
PSG5 for	TACAAAGGACAACTGATGGACC	540	30	57	2.5
PSG5 rev	CTGGGGAGGTCTGGACCAT	540	30	57	2.5
FOXO1a for	GACGCCGTGCTACTCGTT	458	37	55	3
FOXO1a rev	CGGTTCATACCCGAGGTG	458	37	55	3
FOXO3a for	AACCCAGGGCGCTCTTGGTG	281	38	62	3
FOXO3a rev	ATGAGTTCACTACGGATAATGGA	281	38	62	3
FOXO4 for	CCCTCTCAGGAGCCATCA	530	37	55	3
FOXO4 rev	ACCTGGTCCGTAGGGGAG	530	37	55	3
P21 for	CGACTGTGATGCGCTAATGG	482	28	55	1
P21 rev	ACAAACTGAGACTAAGGCAGAAGA	482	28	55	1
NANOG for	TTCCTTCCTCCATGGATCTG	448	35	51	2
NANOG rev	ATTGTTCCAGGTCTGGTTGC	448	35	51	2
TxNIP for	CCTGGTAATTGGCAGCAGATC	199	28	51	2
TxNIP rev	CTTGAGACCATCCATGTCA	199	28	51	2
TP53 for	CCTCCTCAGCATCTTATCCG	259	28	55	1
TP53 rev	GCACAAACACGCACCTCAAA	259	28	55	1
P15 for	CGTTAAGTTTACGGCCAACG	302	43	58	1
P15 rev	GGTAGAGTGGCAGGGTCT	302	43	58	1
P16 for	GGTGCGGGCGCTGCTGGA	210	43	46	1
P16 rev	AGCACCACCAGCGTGTCC	210	43	46	1
P21 for	GGAAGACCATGTGGACCTGT	403	27	58	1
P21 rev	ATGCCCAGCACTCTTAGGAA	403	27	58	1
P27 for	AGTTCGGCTCTGTGAACACC	336	27	58	1
P27 rev	CCACAGTACTGCCACCACAC	336	27	58	1

Primer sequences, fragment lengths and PCR conditions including number of cycles, annealing temperature and magnesium supplementation.

### VDR immunocytochemistry

Human MSC were grown on coverslips in sixwell-plates for three days and then stimulated with 100 nM 1,25D3 for 24 h. Afterwards cells were washed thrice with PBS (pH 7.2), fixed with icecold MetOH (5 min), dried and stored at −80 C until staining. Before and after permeabilization with PBS −0.05% Tween-20, cells were washed thrice with PBS, and then blocked with 3% BSA in PBS. The coverslips were incubated with the primary antibody VDR (C-20), 1∶100, (sc-1008, Santa Cruz Biotechnology, Inc., Heidelberg, Germany) for 16 h at 4°C and a phycoerythrin-labeled secondary antibody, NorthernLights Anti-rabbit IgG-NL557, 1∶400, (NL004, R&D Systems GmbH, Wiesbaden-Nordenstadt, Germany) for 2 h at RT. Finally the coverslips were applied to a drop of Vectashield with DAPI (LINARIS GmbH, Wertheim, Germany) on slides and observed under a fluorescence microscope (Axioskop2, filters 1 and 20, Carl Zeiss MicroImaging GmbH, Göttingen, Germany). Pictures were taken from three independent donors, one representative experiment is shown.

### Immunophenotyping of 1,25D3 treated hMSC: Flow cytometry

Human MSC, cultivated with or without 1,25D3 over 3 passages, were trypsinized, washed and resuspended in PBS plus 1% BSA after P1 and P3. The cells were then labeled with fluorescein isothiocyanate (FITC)- and phycoerythrin (PE)-conjugated monoclonal antibodies. The antibodies used were: CD105-PE, CD90-PE, CD73-PE (mesenchymal markers), HLA-DR-FITC (AbD Serotec, Düsseldorf, Germany) CD34-FITC, CD45-FITC (hematopoietic markers) (BD Biosciences, Heidelberg, Germany). Mouse isotype antibodies served as controls (BD Biosciences, Heidelberg, Germany). Then hMSC were washed twice with PBS plus 1% BSA. For fluorescence activated cell sorting analysis (FACS) labeled cells were resuspended in PBS plus 1% BSA at a concentration of 8×10^5^ cells/ml. Flow cytometry analysis (10000 events) was performed using a BD LSR II (Becton Dickinson, Heidelberg, Germany). Figures were generated using FlowJo software. Analyses were performed by using three independent donors.

### Colony Forming Unit (CFU) Assay

1,25D3 treated hMSC and control hMSC at P1 and P3 respectively were cultured in Petri dishes. The medium was totally renewed every 3–4 days. Fifteen days after standardized seeding at 800 cells per Petri dish (14 cells per cm^2^), cells were washed 2 times with PBS, fixed with methanol, and stained with 0.5% Crystal Violet. Cell clusters of more than 25 cells were counted as CFU originating from one clonal cell. The CFUs were scored using AxioVision Rel. 4.6 (Carl Zeiss MicroImaging GmbH, Göttingen, Germany). Experiments were performed in three to five independent donors.

### Cell proliferation and apoptosis assay

For determination of 1,25D3 short-term effects on proliferation and apoptosis, hMSC (P1 or 2, respectively) were seeded on 96 well plates (1000 cells/well). 24 h after seeding hMSC were stimulated with 100 nM 1,25D3 for 24, 48 and 72 h. Proliferation and apoptosis rates of treated hMSC compared to non-treated hMSC were quantified by Caspase-Glo® 3/7 Assay and CellTiter-Glo® 3/7 Luminescent Cell Viability Assay (both Promega GmbH, Mannheim, Germany) according to the manufacturer's instructions. Luminescence was measured with an Orion II Luminometer (Berthold Detection Systems, Pforzheim, Germany). Proliferation and apoptosis experiments were performed by using hMSC of 3 human donors. Averages were calculated from 3 different measuring points and 3 independent hMSC preparations.

### Growth rate analysis

Passage 0 hMSC were trypsinized and reseeded in culture media with or without 100 nM 1,25D3 at a density of 5000 cells per cm^2^. These hMSC were allowed to grow until they reached 80%–90% confluence and then passaged at the same cell density onwards up to P6. As the cell number of hMSC could be determined for the first time at P1, the cumulative population doubling (CPD) was first determined for P2. CPDs were calculated as described previously [38]–[39].

### Retransformation of 1,25D3 treated hMSC

After three passages of 1,25D3 supplementation, half of these hMSC were cultivated 1,25D3-deprived, whereas the other half was still stimulated. 200 cells per cm^2^ were seeded and after a growth period of two weeks the cell number of 1,25D3 treated and 1,25D3 untreated hMSC was determined.

### Senescence-associated ß-galactosidase staining and determination of ß-galactosidase positive cells

For detection of senescent cells senescence-associated ß-galactosidase staining (counterstain: Nuclear fast red, Merck KGaA, Darmstadt; Germany) was performed as described [24] Cells were observed under the microscope for positive blue stained cytoplasm. Each time five pictures of ß-galactosidase staining of three independent donors were quantified using the AutMess tool of AxioVision Rel. 4.6 software.

### Flow cytometric analysis of oxidative stress

In first-passage and third-passage hMSC cultivated with or without 1,25D3, the accumulation of reactive oxygen species (ROS) was determined using 2′7′-dichlorofluorescin diacetate (H_2_DCF-DA). Intracellular ROS, e.g. hydrogen peroxide, oxidize nonfluorescent H_2_DCF-DA to fluorescent dichlorofluorescin (DCF) [40]–[41]. Cells were incubated with 30 µM H_2_DCF-DA for 45 min, trypsinized, and washed three times with PBS plus 1% BSA. The rate of oxidation was analyzed (3×10^5^ cells/sample) by flow cytometry using a BD LSR I (Becton Dickinson, Heidelberg, Germany) after incubation for 10 min on ice with 1 µg/ml propidium iodide, as described by Schupp et al. [42]. The obtained data were evaluated with the CellQuest Pro software, by defining a region within the DCF-propidium iodide dot blot containing 2×10^3^ propidium iodide-negative cells. The mean value of this population was determined and used for the statistical analysis. Data were obtained from three independent experiments.

### In Vitro Differentiation

Human MSC cultivated with and without 100 nM 1,25D3 for 3 passages were used for differentiation experiments. Differentiation experiments were started in P3 when cells had reached confluence. Human MSC were incubated in expansion medium (negative control) or in cell differentiation medium.

#### Chondrogenic Differentiation

For chondrogenic differentiation hMSC were cultured as high-density pellet cultures. Therefore 2.5×10^5^ cells were resuspended in chondrogenic differentiation medium (DMEM high glucose supplemented with 50 µg/ml ascorbate-2-phosphate, 100 nM dexamethasone, 100 µg/ml pyruvate, 40 µg/ml proline, 1% ITS+1, 1 U/ml penicillin, 100 µg/ml streptomycin and 10 ng/ml TGF-*β*-1, with and without the addition of 100 nM 1,25D3), centrifuged in a 15 ml polypropylene tube (Greiner Bio-One GmbH, Frickenhausen, Germany) at 250 *g* to form a pellet, and cultured for three weeks as described previously [35]. Pellets cultured in chondrogenic differentiation medium without the addition of TGF-*β*-1 served as negative controls. Chondrogenic high-density pellet cultures were fixed in 4% paraformaldehyde, dehydrated through a graded ethanol series and embedded in paraffin. Sections of 4 µm were stained with 1% Alcian Blue (pH 1.0) for sulfated proteoglycans and counterstained with Nuclear Fast Red. Cells cultured in expansion medium served as negative control.

#### Adipogenic Differentiation

For adipogenic differentiation hMSC were seeded at a density of 2.1×10^4^ cells per cm^2^, cultivated until confluence and incubated in adipogenic differentiation medium consisting of DMEM high glucose, 10% FCS, 1 U/ml penicillin, 100 µg/ml streptomycin, 1 µM dexamethasone, 0.5 mM 3-isobutyl-1-methylxanthine (IBMX), 1 µg/ml insulin and 100 µM indomethacin. At day 14 intracellular lipid vesicles of adipogenic monolayer cultures were stained with Oil Red O solution (Merck, Darmstadt, Germany) [43]. During the adipogenic differentiation process, no 1,25D3 was applied. Cells cultured in expansion expansion medium served as negative control.

#### Osteogenic Differentiation

For osteogenic differentiation cells were seeded at a density of 2.1×10^4^ cells per cm^2^ and grown until confluence. Osteogenic differentiation of confluent monolayer cultures was then induced with osteogenic differentiation medium (DMEM high glucose supplemented with 10% FCS, 50 µg/ml ascorbate, 10 mM β-glycerophosphate, 1 U/ml penicillin, 100 µg/ml streptomycin and 100 nM dexamethasone) for four weeks. During the osteogenic differentiation process, no 1,25D3 was applied. Cells cultured in expansion expansion medium served as negative control. The mineralized extracellular matrix was determined by staining for calcium hydrogen phosphate using Alizarin Red S (Chroma-Gesellschaft Scnmid & Co., Stuttgart, Germany) as described previously [44]. Cytoplasmic ALP was stained using the Alkaline Phosphatase, Leukocyte Kit 86-C (Sigma Aldrich GmbH, Munich, Germany) according to the manufacturer's instructions.

### Statistics

The results are expressed as means+standard error (SEM). For comparison between two groups the student's t-test and the Mann-Whitney U test were performed as indicated.

## Supporting Information

Figure S1 **Immunophenotyping of 1,25D3 treated hMSC compared to untreated hMSC.** 1,25D3 treated hMSC and untreated cells at passage 1 (A) and 3 (B) were analyzed with flow cytometry to detect the cell surface marker expression. Results are shown for one representative donor. Yellow peaks represent the specific antibody staining of control cells, blue peaks represent the specific antibody staining of 1,25D3 treated hMSC, and red peaks represent the isotype control antibody staining of 1,25D3 cultured cells (right panels). Histogram overlays show 1,25D3 treated hMSC and control cells gated for CD73, CD90 and CD105 positivity. CD73, CD90 and CD105 expression of 1,25D3 treated cells and untreated hMSC was between 88.9% and 98.7%. This was consistent over three separate experiments using hMSC from three donors (control = 1,25D3 untreated cells; 1,25D3 = 1,25D3 stimulated hMSC; IgG = isotype control).
(TIF)

Figure S2 **Number of CFUs of 1,25D3 treated hMSC.** After passage 1 and 3 the effect of 1,25D3 treatment on CFU number was evaluated in hMSC and compared to untreated hMSC. No significant difference was obtained in CFU number between 1,25D3 treated cells (gray bars) and control hMSC (black bars) at P1 and also at P3. Data show mean+SEM of three (P3) or five (P1) independent experiments, respectively.
(TIF)

Figure S3 **Retransformation of 1,25D3 stimulated hMSC.** **A** The 1,25D3 induced morphological changes are not completely reversible when 1,25D3 treated hMSC are again cultured in normal medium. Some cells retrained their 1,25D3 induced enlarged volume (r.c. = retransformed cells; 1,25D3 = 1,25D3 treated hMSC). The results are shown for one representative donor and observations were consistent over 3 independent experiments using hMSC from three donors. Scale bar = 50 µm. **B** The cell number of 1,25D3 treated and retransformed cells was determined. Data show mean+SEM of three independent experiments using cells from three donors. **C** The cell number of 1,25D3 treated and retransformed cells was determined. Therefore three donors were analyzed. Retransformed hMSC displayed an obvious higher cell number compared to 1,25D3 treated cells
(TIF)

Figure S4 **Osteogenic differentiation capacity of 1,25D3 treated hMSC.** **A** Staining for ALP after osteogenic differentiation for 14 days (upper row) and for 28 days (lower row). Staining for cytoplasmic ALP was performed for undifferentiated hMSC (n.d.) as well as for hMSC pretreated with and without 1,25D3 (C = differentiated control; +1,25D3 = differentiated 1,25D3 pretreated cells). The figure is representative of three independent experiments. Scale bar = 50 µm. **B** Quantification of ALP staining after 1,25D3 pretreatment over 3 passages and 1,25D3 untreated hMSC over 3 passages as a result of two or four weeks of osteogenic differentiation. The Fold Change was calculated by comparing the induction of differentiation of 1,25D3 pretreated cells with control cells. After two weeks under osteogenic conditions, 1,25D3 pretreated hMSC showed a slightly enhanced ALP staining compared to untreated cells, and after four weeks under osteogenic conditions, 1,25D3 pretreated hMSC revealed significant reduced ALP staining compared to untreated hMSC (*; p<0.05, student's t-test). The results are shown as means of three independent experiments+SEM using different preparations of hMSC. Each time 6 pictures of ALP staining were analyzed using the AutMess tool of AxioVision Rel. 4.6 software.
(TIF)

Figure S5 **Flow cytometric analysis of 1,25D3-induced ROS formation in hMSC after P1 and P3.** Oxidative stress in cells cultured for one passage (**A**) and for three passages (**B**) with (gray bar) and without (black bar) 1,25D3. The results are shown as mean+SEM of three independent experiments, each normalized to its control and performed in triplicates. Cells from four different donors were used (*, p<0.05, Mann-Whitney U test).
(TIF)
