# Supplementary figures and images for: 1,25-Dihydroxyvitamin D3 Treatment Delays Cellular Aging in Human Mesenchymal Stem Cells while Maintaining Their Multipotent Capacity
Source: PLoS One. 2012 Jan 5;7(1):e29959. doi: 10.1371/journal.pone.0029959 (PMC3252365; doi:10.1371/journal.pone.0029959)

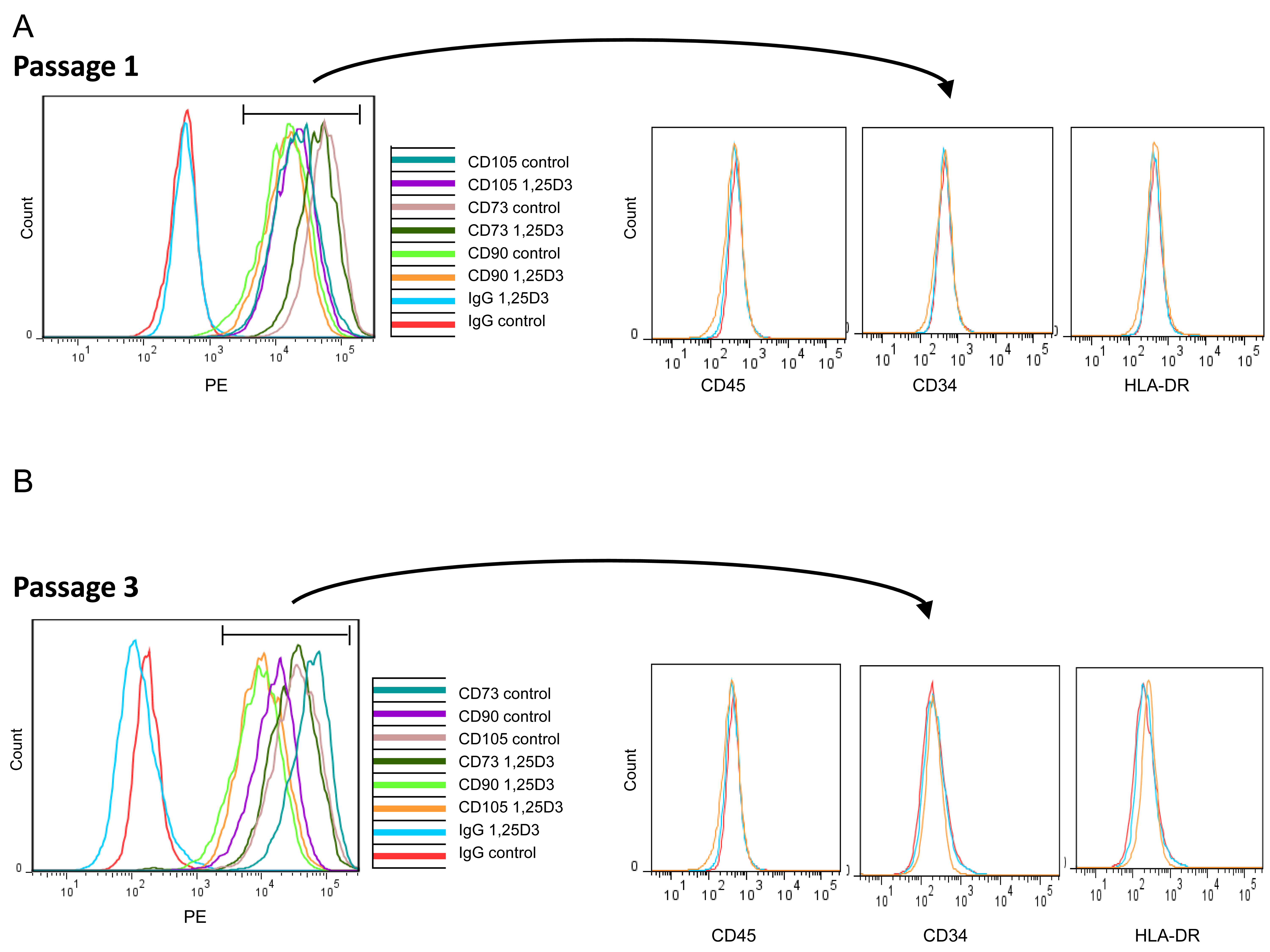

Supplement: Figure S1 — Immunophenotyping of 1,25D3 treated hMSC compared to untreated hMSC. 1,25D3 treated hMSC and untreated cells at passage 1 (A) and 3 (B) were analyzed with flow cytometry to detect the cell surface marker expression. Results are shown for one representative donor. Yellow peaks represent the specific antibody staining of control cells, blue peaks represent the specific antibody staining of 1,25D3 treated hMSC, and red peaks represent the isotype control antibody staining of 1,25D3 cultured cells (right panels). Histogram overlays show 1,25D3 treated hMSC and control cells gated for CD73, CD90 and CD105 positivity. CD73, CD90 and CD105 expression of 1,25D3 treated cells and untreated hMSC was between 88.9% and 98.7%. This was consistent over three separate experiments using hMSC from three donors (control = 1,25D3 untreated cells; 1,25D3 = 1,25D3 stimulated hMSC; IgG = isotype control). (TIF) [file pone.0029959.s001.tif]

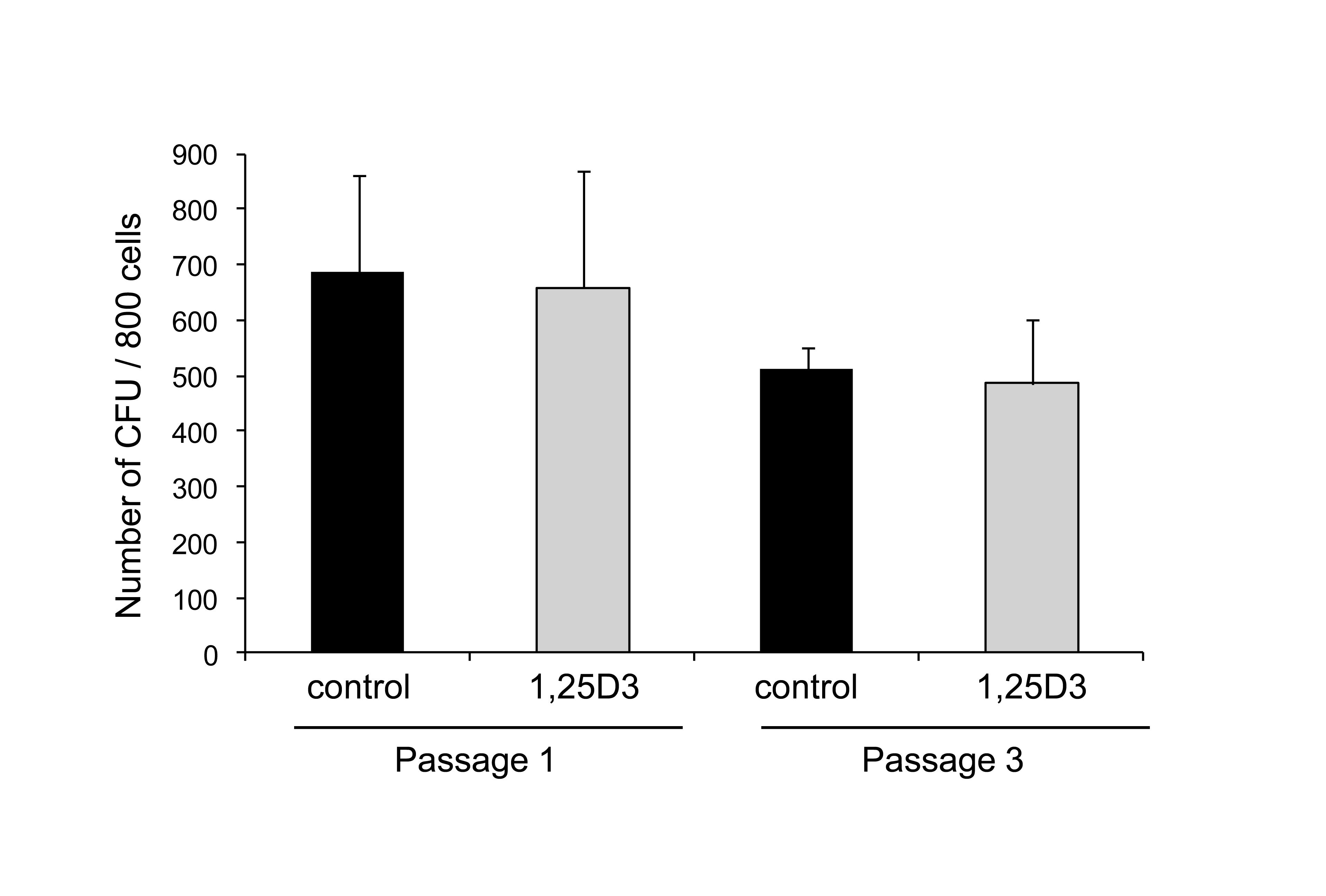

Supplement: Figure S2 — Number of CFUs of 1,25D3 treated hMSC. After passage 1 and 3 the effect of 1,25D3 treatment on CFU number was evaluated in hMSC and compared to untreated hMSC. No significant difference was obtained in CFU number between 1,25D3 treated cells (gray bars) and control hMSC (black bars) at P1 and also at P3. Data show mean+SEM of three (P3) or five (P1) independent experiments, respectively. (TIF) [file pone.0029959.s002.tif]

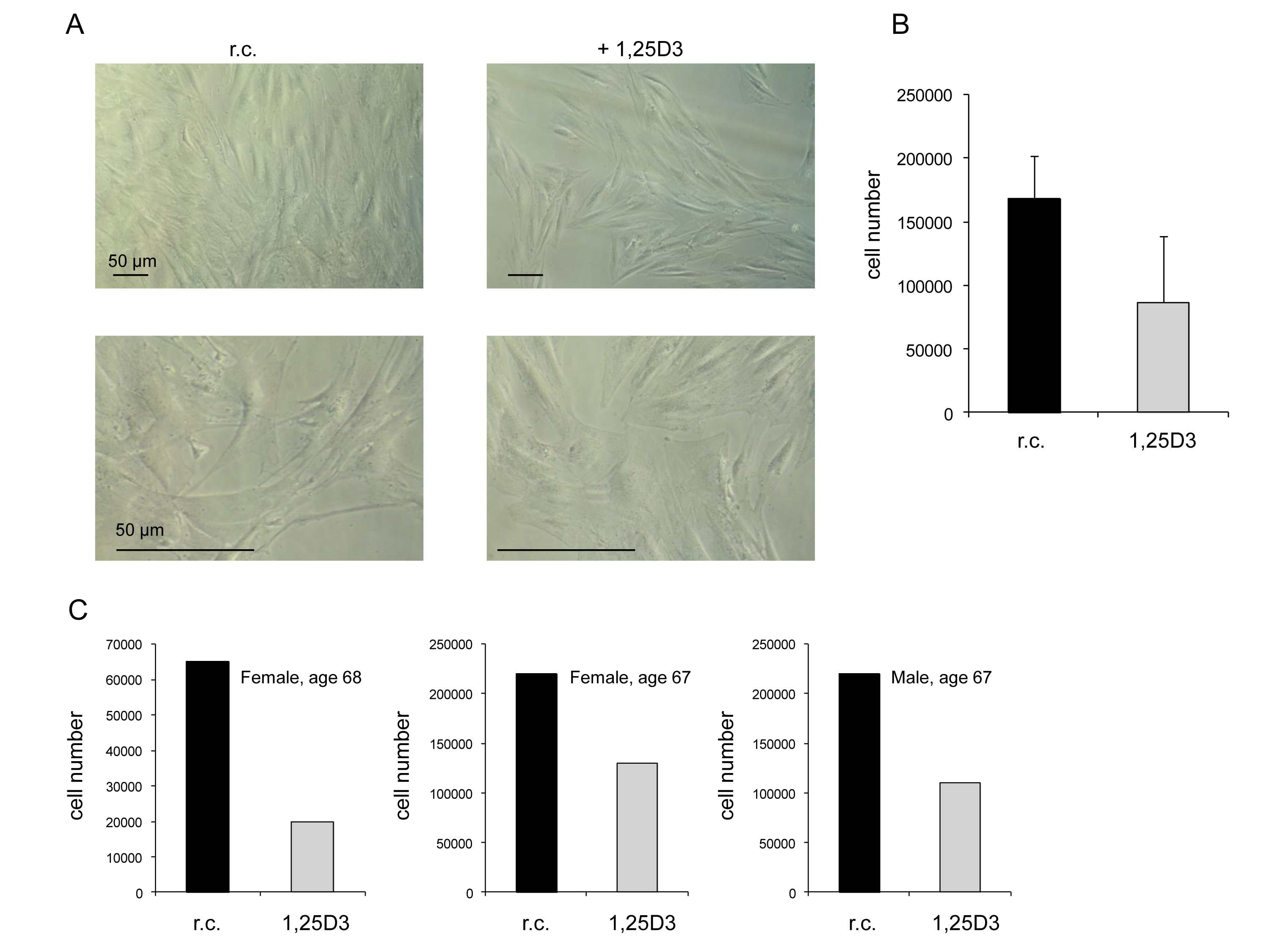

Supplement: Figure S3 — Retransformation of 1,25D3 stimulated hMSC. A The 1,25D3 induced morphological changes are not completely reversible when 1,25D3 treated hMSC are again cultured in normal medium. Some cells retrained their 1,25D3 induced enlarged volume (r.c. = retransformed cells; 1,25D3 = 1,25D3 treated hMSC). The results are shown for one representative donor and observations were consistent over 3 independent experiments using hMSC from three donors. Scale bar = 50 µm. B The cell number of 1,25D3 treated and retransformed cells was determined. Data show mean+SEM of three independent experiments using cells from three donors. C The cell number of 1,25D3 treated and retransformed cells was determined. Therefore three donors were analyzed. Retransformed hMSC displayed an obvious higher cell number compared to 1,25D3 treated cells (TIF) [file pone.0029959.s003.tif]

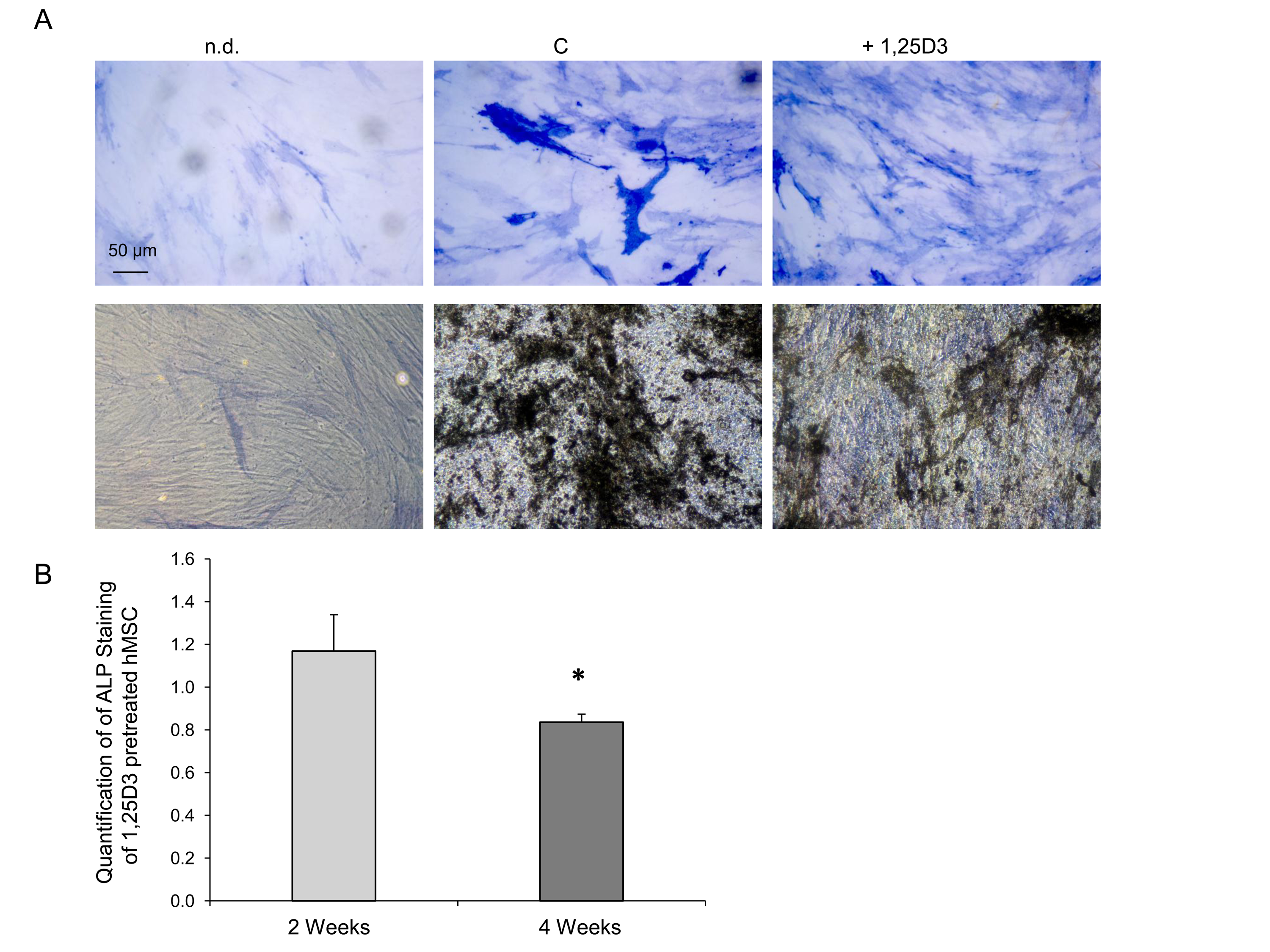

Supplement: Figure S4 — Osteogenic differentiation capacity of 1,25D3 treated hMSC. A Staining for ALP after osteogenic differentiation for 14 days (upper row) and for 28 days (lower row). Staining for cytoplasmic ALP was performed for undifferentiated hMSC (n.d.) as well as for hMSC pretreated with and without 1,25D3 (C = differentiated control; +1,25D3 = differentiated 1,25D3 pretreated cells). The figure is representative of three independent experiments. Scale bar = 50 µm. B Quantification of ALP staining after 1,25D3 pretreatment over 3 passages and 1,25D3 untreated hMSC over 3 passages as a result of two or four weeks of osteogenic differentiation. The Fold Change was calculated by comparing the induction of differentiation of 1,25D3 pretreated cells with control cells. After two weeks under osteogenic conditions, 1,25D3 pretreated hMSC showed a slightly enhanced ALP staining compared to untreated cells, and after four weeks under osteogenic conditions, 1,25D3 pretreated hMSC revealed significant reduced ALP staining compared to untreated hMSC (*; p<0.05, student's t-test). The results are shown as means of three independent experiments+SEM using different preparations of hMSC. Each time 6 pictures of ALP staining were analyzed using the AutMess tool of AxioVision Rel. 4.6 software. (TIF) [file pone.0029959.s004.tif]

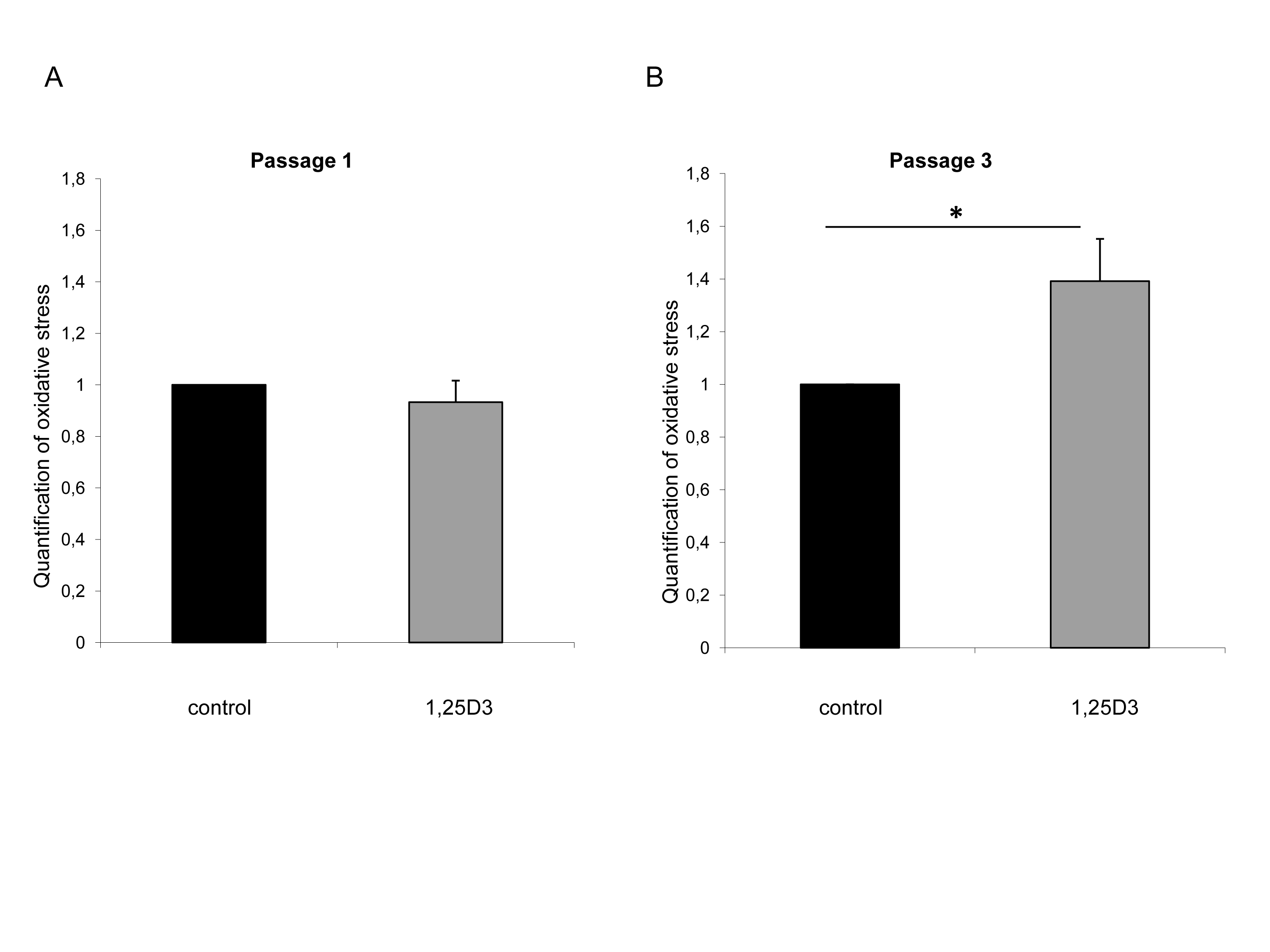

Supplement: Figure S5 — Flow cytometric analysis of 1,25D3-induced ROS formation in hMSC after P1 and P3. Oxidative stress in cells cultured for one passage (A) and for three passages (B) with (gray bar) and without (black bar) 1,25D3. The results are shown as mean+SEM of three independent experiments, each normalized to its control and performed in triplicates. Cells from four different donors were used (*, p<0.05, Mann-Whitney U test). (TIF) [file pone.0029959.s005.tif]
